# Glucose Intolerance after a Recent History of Gestational Diabetes

**DOI:** 10.1155/2014/727652

**Published:** 2014-08-07

**Authors:** Katrien Benhalima, Liesbeth Leuridan, Peggy Calewaert, Roland Devlieger, Johan Verhaeghe, Chantal Mathieu

**Affiliations:** ^1^Department of Endocrinology, UZ Gasthuisberg, KU Leuven, Herestraat 49, 3000 Leuven, Belgium; ^2^Department of Obstetrics & Gynecology, UZ Gasthuisberg, KU Leuven, Herestraat 49, 3000 Leuven, Belgium

## Abstract

*Aim*. Our aim was to evaluate the uptake of our current screening strategy postpartum and the risk factors for glucose intolerance in women with a recent history of gestational diabetes (GDM). *Methods*. Retrospective analysis of files of women with a recent history of GDM diagnosed with the Carpenter and Coustan criteria from 01-01-2010 till 31-12-2013. Multivariable logistic regression was used to adjust for confounders. *Results*. Of all 231 women with a recent history of GDM, 21.4% (46) did not attend the scheduled postpartum OGTT. Of the women tested, 39.1% (66) had glucose intolerance and 5.3% (9) had diabetes. These women were more often overweight (39.7% versus 25.3%, *P* = 0.009), were more often treated with basal-bolus insulin injections (52.0% versus 17.4%, *P* = 0.032), and had a lower beta-cell function and lower insulin sensitivity, remaining significant after adjustment for age, BMI, and ethnicity (insulin secretion sensitivity index-2 (ISSI-2) in pregnancy 1.5 ± 0.5 versus 1.7 ± 0.4, *P* = 0.029; ISSI-2 postpartum 1.5 (1.2–1.9) versus 2.2 (1.8–2.6), *P* = 0.020; Matsuda index postpartum 3.8 (2.6–6.2) versus 6.0 (4.3–8.8), *P* = 0.021). *Conclusion*. Glucose intolerance is frequent in early postpartum and these women have a lower beta-cell function and lower insulin sensitivity. One fifth of women did not attend the scheduled OGTT postpartum.

## 1. Introduction

Gestational diabetes (GDM) is a frequent medical condition during pregnancy and was historically defined as “any degree of glucose intolerance with onset or first recognition during pregnancy” [1]. Internationally, the best approach for screening and diagnosis of GDM remains controversial. The “International Association of Diabetes and Pregnancy Study Groups” (IADPSG) recommends a one-step screening strategy with the 2-hour 75 g oral glucose tolerance test (OGTT) using stricter criteria to diagnose GDM [2]. The latest 2014 American Diabetes Association (ADA) recommendations leave open the option between the one-step IADPSG recommendation and the two-step screening strategy with a glucose challenge test (GCT) [3]. Using the IADPSG criteria on the “Hyperglycemia and Adverse Pregnancy Outcomes” (HAPO) cohort, GDM prevalence varied from 9.3% to >25% depending on the populations studied [4].

GDM is associated with an increased risk for a large for gestational age (LGA) baby, which in turn increases risks of shoulder dystocia and caesarian deliveries [5]. When the baby is female, exposure to maternal hyperglycaemia in utero might increase her own risk of subsequently developing GDM in her own pregnancies [6]. Shortly after delivery, the glucose values are generally restored to normal, but women with GDM have a sevenfold increased risk of developing T2DM [7]. The risk of women with GDM, diagnosed with the Carpenter and Coustan criteria, to develop T2DM postpartum depends on the populations studied and is generally around 30–50% within 10 years after the index pregnancy [7]. The best postpartum screening strategy for glucose intolerance among women with a history of GDM is still debated. The ADA now recommends to screen women with a history of GDM at 6–12-week postpartum using the 2 h 75 g oral glucose tolerance test (OGTT) and nonpregnancy diagnostic criteria but this is mostly based on expert consensus or clinical experience [3]. The ADA further advises that these women have lifelong screening for the development of T2DM and prediabetes at least every 3 years, by using Hba1c, FPG, or the 2 h 75 g OGTT [3]. In routine clinical care, postpartum testing in women with a previous history of GDM rates often remains around 50% [8].

More data are necessary in different populations on the risk factors, both clinical and biochemical, to develop glucose intolerance in early postpartum. The aim of our study was to evaluate the uptake of our current screening strategy postpartum and to determine the prevalence and the risk factors for early postpartum glucose intolerance in women with a recent history of GDM.

## 2. Subjects and Methods

Retrospective analysis of the electronic medical files of the University Hospital UZ Leuven in Belgium, from 01-01-2010 till 31-12-2013 of all women with a recent history of GDM diagnosed with the Carpenter and Coustan criteria. The study was approved by the Institutional Review Board of UZ Leuven (ML 10085).

Approximately 2400 women are delivered annually at our hospital. The background prevalence of T2DM in Belgium is 5.3% [9]. In the general adult population, 28% of women are overweight and 13% are obese [10]. Leuven is a medium size city in the region of Flanders and has a population with a rather low background number of women from ethnic minorities (BME) (10.9%) [10]. Accurate data on the prevalence of GDM are lacking in Belgium and the current practice for screening for GDM varies across different centers [11]. The lack of consensus on screening for GDM is also apparent in the two linguistic regions of Belgium: a recent Flemish consensus between endocrinologists, gynecologists, and general physicians advises at this moment to continue with the two-step screening strategy while the recent consensus of the French-speaking obstetricians is to adopt the IADPSG strategy for GDM [12, 13].

Women are not yet universally screened for overt diabetes early in pregnancy in our hospital. All pregnant women were screened and diagnosed for GDM according to the Fifth International Workshop Conference criteria [14]. Women received a GCT and those testing positive (threshold after 1 h ≥ 140 mg/dL (7.8 mmol/L)) had a 3 h 100 g OGTT at 24–28 gestational weeks using the Carpenter and Coustan criteria for GDM (FPG ≥ 95 mg/dL (5.3 mmol/L), 1 h glycaemia ≥ 180 mg/dL (10.0 mmol/L), 2 h glycaemia ≥ 155 mg/dL (8.6 mmol/L), and 3 h glycaemia ≥ 140 mg/dL (7.8 mmol/L), diagnosis of GDM if ≥2 values are abnormal). Previous research from our group showed that the GDM prevalence in our hospital is 3.3% using the Carpenter and Coustan criteria [15, 16]. Women with GDM were treated with insulin 1-2 weeks after the implementation of dietary measures when FPG ≥ 95 mg/dL (5.3 mmol/L) and/or 2 h postprandial glycaemia ≥ 120 mg/dL (6.7 mmol/L). In our center, oral antidiabetes drugs are not routinely used during pregnancy. In UZ Leuven, since January 2010, every woman with a previous diagnosis of GDM is advised to receive a 2 h 75 g OGTT 3 months after the delivery. Before 2010, only insulin-treated women with GDM received an 75 g OGTT postpartum in our center. There are no data in our database on how long or how exclusive breastfeeding was given. There are no general recommendations on breastfeeding and OGTT postpartum in Belgium but the general recommendation in our hospital is to postpone the OGTT to 6-month postpartum if women are still breastfeeding at 3 months. If women are still breastfeeding at 6 months, the OGTT is performed despite the breastfeeding.

Outcomes were obtained from review of the electronic database. Maternal characteristics recorded were age, weight, body mass index (BMI) at first prenatal visit and at delivery, overweight (BMI ≥ 25 Kg/m²), obesity (BMI ≥ 30 Kg/m²), weight gain (difference in weight between first prenatal visit and the delivery), ethnicity, parity, smoking and alcohol habits, family history of diabetes, history of GDM, history of polycystic ovary syndrome (PCOS), hypertension, dyslipidemia, and glucose intolerance before pregnancy. Excessive weight gain was defined according to the most recent Institute of Medicine (IOM) guidelines, except for obese women for whom we use the recommendation to gain ≤5 Kg during pregnancy [17]. Other data that were recorded are whether or not the scheduled postpartum OGTT was attended, the timing of the OGTT postpartum, the glucose values and the insulin values based on the 75 g OGTT postpartum (0 min-30 min-60 min-120 min) and based on the 100 g OGTT during pregnancy (0 min-60 min-120 min-180 min), whether women breastfed or not, type of contraception used postpartum, gestational age at delivery, the timing and result of the GCT, the gestational age at the diagnosis of GDM, HbA1c at the time of the 100 g OGTT during pregnancy, whether women received treatment with corticoids during pregnancy after the screening test, need of insulin, type of insulin and number of injections, and the gestational age at the start of insulin.

Maternal outcomes recorded were diabetes postpartum (FPG ≥ 126 mg/dL (7.0 mmol/L) and/or 2 hour glycaemia at the OGTT postpartum ≥ 200 mg/dL (11.1 mmol/L)) and glucose intolerance postpartum (impaired fasting glucose with a FPG ≥100–125 mg/dL (5.5–6.9 mmol/L) and/or impaired glucose tolerance with a 2-hour glycaemia at the OGTT postpartum ≥ 140–199 mg/dL (7.8–11.0 mmol/L)) [3].

Insulin sensitivity was measured using the insulin sensitivity index of Matsuda, a well-established measure of whole-body insulin sensitivity [18]. The insulin sensitivity index of Matsuda is defined as 10 000/  ((FPG × fasting plasma insulin) × (mean glucose during OGTT × mean insulin during OGTT)) [18]. As a secondary measure of insulin sensitivity (largely hepatic), we also calculated the reciprocal of the homeostasis model assessment of insulin resistance (1/HOMA-IR) [19]. HOMA-IR is calculated as the product of FPG and fasting plasma insulin divided by 22.5 [19]. Beta-cell function was assessed by the insulinogenic index dived by HOMA-IR. The insulinogenic index was calculated as the incremental change in insulin concentration during the first 30 min of the OGTT divided by the incremental change in glucose during the same period [20, 21]. As a secondary measure of beta-cell function, the insulin secretion sensitivity index (ISSI-2) was measured, an OGTT-derived measure that is analogous to the disposition index obtained from the frequently sampled intravenous glucose tolerance test [22, 23]. Glycaemia was assessed by the area under the glucose curve during the OGTT, calculated using the trapezoidal rule [22, 23]. ISSI-2 is defined as the product of (1) insulin secretion measured by the ratio of the area under the insulin curve to the area under the glucose curve and (2) insulin sensitivity measured by the insulin sensitivity index of Matsuda [22, 23]. All these measures have been validated for use in pregnancy.

HbA_1c_ was measured by reversed-phase cation-exchange chromatography (ADAMS HA-8160, Menarini Diagnostics Benelux, Zaventem, Belgium). Plasma glucose was measured by an automated colorimetric-enzymatic method (hexokinase-glucose-6-phosphate-dehydrogenase, application 668) on a Hitachi/Roche-Modular P analyzer. Insulin was measured by the immunometric ECLIA (Roche Modular E170, Basel, Switzerland).

*Statistical Analyses*. Statistical analyses were performed using SPSS 22.0. Continuous data were expressed as mean and standard deviation if normally distributed; nonparametric variables were expressed as median. Categorical data were expressed as percentage. To compare variables between two groups, independent samples *t*-tests were used for normally distributed continuous variables, Mann-Whitney *U*-test for nonparametric variables, and chi-squared tests for categorical variables. Multivariable logistic regression was used to analyse the impact of possible confounders such as age, BMI, ethnicity, breastfeeding, contraception, multiparity, and corticoid treatment on insulin sensitivity and beta-cell function. A *P* value of <0.05 (two-tailed) was considered significant.

## 3. Results

Over a 4-year period, 255 women were identified with a recent history of GDM. After evaluation of the medical files, 24 files were not included in the analysis due to insufficient data (9), because women only had one abnormal value on the OGTT (9) or because women took part in a study using the IADPSG criteria for GDM (6), leaving a cohort of 231 women with a recent history of GDM for analysis. Of all the 231 women, 16 women did not receive an appointment in our hospital for the postpartum OGTT due to the diagnosis of T2DM (6) or MODY-2 (1) early after the delivery or because the postpartum OGTT was planned in another local hospital (9).

The mean age of the cohort was 32.3 years (±4.8), 12.6% (29) had a previous history of GDM, 36.4% (84) had a BME background, 34.2% (75) were overweight, and 23.3% (51) were obese at the first prenatal visit. The most frequent BME background was Northern-African (10.0%), Black African (7.8%), South Asian (11.3%), and Middle East (4.3%). The median week at diagnosis of GDM was 27.0 (25.0–29.0) and 29.6% (68) of women needed insulin during pregnancy. Of all women, 71.4% (165) were breastfeeding.

Of all women (215) who received an appointment for a postpartum OGTT, 21.4% (46) did not attend the scheduled OGTT. Moreover, 10.7% (20) of all OGTT's postpartum were only performed after a telephonic recall for a new appointment after missing the first scheduled OGTT. Compared to women who received an OGTT postpartum, women who did not attend the postpartum OGTT had more often a previous history of smoking before pregnancy (10.9% versus 2.4%, *P* = 0.021), had more often a previous history of GDM (21.7% versus 10.7%, *P* = 0.042), were less often breastfeeding (56.5% versus 75.7%, *P* = 0.011), and had a significant lower insulin sensitivity based on the OGTT during pregnancy (Table 1). The postpartum OGTT was performed at a median of 12 weeks (8–13) with a minimum of 6 weeks. The OGTT was performed at the latest at six months postpartum expect for one woman at 32 weeks.

Of all women (169) receiving an OGTT postpartum, 39.1% (66) had glucose intolerance of which 21.2% (14/66) had impaired fasting glucose (IFG), 66.7% (44/66) had impaired glucose tolerance (IFG), and 12.1% (8/66) had IFT/IGT combined. Of all women (169) receiving an OGTT postpartum, 5.3% (9) had diabetes: one women had diabetes based on the FPG (173 mg/dL (9.6 mmol/L)) and 8 women had diabetes based on the 2-hour glycaemia on the OGTT (values ranging from 206 to 288 mg/dL (11.4–16.0 mmol/L)). Compared to women with a normal OGTT postpartum, women with glucose intolerance (IFG and/or IGT) or diabetes were more often overweight (39.7% versus 25.3%, *P* = 0.009) or obese at first prenatal visit (27.4% versus 19.5%, *P* = 0.037), were more often multiparous (44.0% versus 26.6%, *P* = 0.028), had a higher glucose value after the GCT (168.0 mg/dL (153.0–190.0) versus 159.0 mg/dL (149.5–175.5), *P* = 0.007), had an earlier diagnosis of GDM (gestational weeks 26.0 (25.0–28.0) versus 27.0 (25.0–29.0), *P* = 0.030), had a higher median fasting (94.5 mg/dL (84.2–101.7) versus 88.0 mg/dL (81.0–99.0), *P* = 0.006) and 2-hour glucose value on the OGTT during pregnancy (175.0 mg/dL (162.0–198.0) versus 168.0 mg/dL (158.0–181.0), *P* = 0.003), and were more often treated with basal-bolus insulin injections (52.0% versus 17.4%, *P* = 0.032) (Table 2). Women with glucose intolerance or diabetes postpartum also had a significant impaired beta-cell function and lower insulin sensitivity, remaining significant after adjustment for age, BMI, ethnicity, breastfeeding, contraception, multiparity, and corticoid treatment (Table 3). The data also remained significantly different when the 9 women with diabetes were not included in the analysis.

Compared to women with IGT based on the 2-hour glucose value postpartum, women with IFG postpartum were more often obese (50% versus 14.3%, *P* = 0.006), had an earlier diagnosis of GDM (gestational weeks 25.0 (20.5–27.0) versus 27.0 (25.0–28.7), *P* = 0.029), had a higher median fasting (99.5 mg/dL (96.2–121.0) versus 89.0 mg/dL (84.0–101.0), *P* = 0.022) and 1-hour glucose value on the OGTT during pregnancy (202.5 mg/dL (183.0–226.2) versus 189.0 mg/dL (170.0–202.0), *P* = 0.047), had more often four abnormal glucose values on the OGTT during pregnancy (41.7% versus 14.0%, *P* = 0.034), and insulin was started earlier during pregnancy (gestational weeks 24.0 (20.7–28.2) versus 30.0 (27.5–32.0), *P* = 0.017)) (Table 4). Women (8) with the combination of IFG and IGT were excluded from these analyses. Compared to women with IGT postpartum, women with IFG also had an impaired beta-cell function and lower insulin sensitivity based on the OGTT postpartum (Table 5). However, after adjustment for the BMI at first prenatal visit, only 1/HOMA-IR remained significant (*P* = 0.016).

## 4. Discussion

Our study shows that glucose intolerance is very frequent in women with a recent history of GDM. In our study, 44.4% of all women had glucose intolerance or diabetes in early postpartum. Of all women, 39.1% had glucose intolerance of which 66.7% had IGT, 21.2% had IFG, and 12.1% had IFG and IGT combined. A FPG alone postpartum would therefore have missed the majority of women with glucose intolerance, confirming the need for an OGTT in early postpartum in our population. Studies evaluating the use of HbA1c alone or in combination with FPG to diagnose glucose intolerance in women who have had GDM show conflicting results with sensitivity rates of HbA1c and FPG combined ranging from 83.0% to 90.0% [24, 25]. We have no data on HbA1c postpartum in our cohort. The high prevalence of glucose intolerance postpartum in our cohort is probably related to the use of the Carpenter and Coustan criteria for GDM identifying women at high risk for the development of diabetes. The use of the IADPSG criteria for GDM will result in a greater proportion of women diagnosed with GDM but this will presumably lead to a lower proportion at risk for postpartum glucose intolerance [26].

The most important risk factors to develop glucose intolerance postpartum differ according to the populations studied. A recent study showed that a metabolic-syndrome-like cluster in pregnant GDM women is an important predictor of the 10-year risk for T2DM [27]. In a Korean population, independent risk factors for the risk to develop T2DM in early postpartum were a higher prepregnancy BMI, higher area under the curve of glucose during an antepartum OGTT, lower fasting insulin concentration, and a decreased beta-cell function [28]. The most important risk factors in our population to develop glucose intolerance or diabetes postpartum were a higher BMI at first prenatal visit, higher glucose values on the GCT and OGTT during pregnancy, an earlier diagnosis of GDM, and the need for bolus-basal insulin injections. Breastfeeding has been associated with lower FPG and insulin, and a lower prevalence of glucose intolerance 6–9-week postpartum [29, 30]. Breastfeeding was very prevalent in our cohort of women who received an OGTT postpartum and rates were not lower in women with glucose intolerance postpartum. Maternal age at diagnosis is often considered a significant risk factor for glucose intolerance in the future but this was not a risk factor in our population [31, 32]. This is probably due to the high mean age of our cohort and the short follow-up after the delivery.

We also show that women with glucose intolerance or diabetes postpartum have an impaired beta-cell function and lower insulin sensitivity, remaining significant after adjustment for age, BMI, ethnicity, breastfeeding, contraception, multiparity, and corticoid treatment. Our data therefore highlight that variations in underlying maternal insulin secretion and sensitivity together with the degree of metabolic stress posed by factors specific to the pregnancy, all contribute to variations in postpartum risk for glucose intolerance.

Our data also show that women with IFG have a more adverse risk profile compared to women with IGT. The lower insulin sensitivity and lower beta-cell function in women with IFG seems to be largely driven by a higher BMI. IFG and IGT clearly represent different underlying deteriorations in glucose metabolism. Previous investigations have proposed that subjects with isolated IFG suffer from impaired first-phase insulin secretion and increased hepatic insulin resistance [33]. It has also been shown that when measurements of beta-cell function were adjusted for severity of insulin resistance, subjects with IGT and combined IFG/IGT had a significantly greater reduction in insulin secretion than subjects with IFG [34]. This is confirmed by our data showing that after adjustment for the BMI at first prenatal visit, only 1/HOMA-IR remained significant in subjects with IFG. In contrast, impaired muscle insulin sensitivity is rather reflected by elevated 2 h glucose levels resulting in IGT [33, 34].

In our study, one fifth of women did not attend the scheduled OGTT postpartum. These women have an adverse risk profile compared to women who attended the scheduled OGTT postpartum. A telephonic recall for a new appointment for the postpartum OGTT increased the screening uptake with 10% in our cohort but more efforts are clearly necessary. In routine clinical care, reported postpartum testing rates are even lower with only 30–50% of women with recent GDM who receive an OGTT within 6 months after the delivery [35–37]. Annual follow-up rates are often even lower [38]. This is a missed opportunity in a high-risk population to timely detect glucose intolerance and start lifestyle interventions to prevent or delay the development of glucose intolerance and diabetes [39]. Other systems to improve early postpartum diabetes screening among women with GDM such as an electronic system to trigger reminder calls or postpartum SMS reminders might be helpful tools [40, 41]. In a Southern Italian cohort of women with GDM, verbal and written counseling on the follow-up significantly increased the adherence rate [42]. In this study, the most compliant ones were older women, women with a previous history of GDM, higher educational levels, overweight or obese women, and those with insulin treatment. This is in contrast with the results of our study showing that women who failed to attend the postpartum OGTT had more often a previous history of GDM. More efforts are therefore necessary to better inform and create a greater awareness among these women of their future risk for developing diabetes. To better organize the long term follow-up to timely screen for diabetes in women with a history of GDM, in our center women are also invited to register in the Flemish project “Zoet Zwanger” (Sweet Pregnancy). This project is an initiative of the Flemish Diabetes Association and supported by the Flemish government, whereby women receive yearly remainders to have the FPG checked by their general practitioner [43].

Strengths of the study are the detailed characterization of a relatively large cohort of women with recent GDM using a good database. Multiple measures of beta-cell function and insulin sensitivity were calculated and adjusted for confounders. A limit of the study is the retrospective nature of the analysis and the lack of longer term data postpartum on the risk for glucose intolerance.

In conclusion, we show that glucose intolerance (IFG, IGT, and diabetes) is frequent in early postpartum and these women have an impaired beta-cell function and lower insulin sensitivity. One fifth of women did not attend the scheduled OGTT postpartum and these women have an adverse risk profile compared to women who received the OGTT postpartum.

## Figures and Tables

**Table 1:** Comparison of the characteristics between women who received an OGTT postpartum and women who failed to attend the scheduled OGTT postpartum.

	Women with OGTT postpartum *N* = 169 (78.6%)	Women without OGTT postpartum *N* = 46 (21.4%)	*P* value
Age mean years	32.2 ± 4.7	31.5 ± 4.9	0.349
% overweight at first prenatal visit	31.9	40.9	0.195
% obese at first prenatal visit	23.1	25.0	0.795
% BME	33.7	37.0	0.683
% smoking before pregnancy	2.4	10.9	**0.021**
% first degree family member with T2DM	15.4	13.0	0.580
% history of GDM	10.7	21.7	**0.042**
% multiparous	34.3	34.8	0.872
% breastfeeding	75.7	56.5	**0.011**
Week OGTT (median)	27.0 (25.0–28.2)	27.0 (25.0–29.0)	0.520
% fasting abnormal	42.3	45.0	0.759
% ≥4 values abnormal on the 100 g OGTT	13.5	17.9	0.475
% insulin	28.6	26.1	0.818
Weeks start insulin (median)	29.5 (27.0–32.0)	30.0 (27.0–33.0)	0.582
% bolus-basal injections	35.4	33.3	0.786
pISSI-2 mean	1.6 ± 0.4	1.6 ± 0.6	0.690
pMatsuda (median)	2.8 (1.9–3.8)	2.2 (1.5–3.1)	**0.033**
p1/HOMA-IR (median)	0.020 (0.012–0.032)	0.015 (0.010–0.023)	**0.030**

BME: ethnic minority backgrounds; T2DM: type 2 diabetes; GDM: gestational diabetes; OGTT: oral glucose tolerance test; pISSI-2: insulin secretion sensitivity index during pregnancy; pMatsuda: insulin sensitivity index of Matsuda during pregnancy; p1/HOMA-IR: the reciprocal of the homeostasis model assessment of insulin resistance during pregnancy.

**Table 2:** Comparison of the characteristics between women with a normal OGTT and women with glucose intolerance/diabetes postpartum.

	Normal *N* = 94 (55.6%)	Glucose intolerance/diabetes *N* = 75 (44.4%)	*P* value
Age mean years	31.8 ± 4.8	32.9 ± 4.6	0.141
BMI kg/m² at first prenatal visit (median)	24.1 (21.5–28.9)	27.8 (22.9–30.7)	**0.019**
% overweight at first prenatal visit	25.3	39.7	**0.009**
% obese at first prenatal visit	19.5	27.4	**0.037**
% excessive weight gain	31.3	23.0	0.241
% BME	24.5	45.3	**0.004**
% smoking before pregnancy	3.2	1.3	0.713
% first degree family member with T2DM	16.0	14.7	0.095
% history of GDM	5.3	17.3	0.016
% history of PCOS	2.1	2.7	0.905
% history of hypertension	3.2	5.3	0.569
% history of dyslipidaemia	3.2	1.3	0.261
% multipareous	26.6	44.0	**0.028**
% breastfeeding	77.7	73.0	0.536
% progestin-only oral contraceptive	62.5	67.8	0.527
Result GCT mg/dL (median)	159.0 (149.5–175.5)	168.0 (153.0–190.0)	**0.007**
Week at diagnosis of GDM (median)	27.0 (25.0–29.0)	26.0 (25.0–28.0)	**0.030**
% ≥4 values abnormal on 100 g OGTT	9.1	19.1	0.069
Median fasting 100 g OGTT mg/dL	88.0 (81.0–99.0)	94.5 (84.2–101.7)	**0.006**
Median 1 h 100 g OGTT mg/dL	188.0 (167.0–199.0)	191.5 (170.2–205.5)	0.312
Median 2 h 100 g OGTT mg/dL	168.0 (158.0–181.0)	175.0 (162.0–198.0)	**0.003**
Median 3 h 100 g OGTT mg/dL	144.0 (122.0–159.0)	151.5 (128.5–166.0)	0.058
HbA1c % (median)	5.3 (5.1–5.5)	5.4 (5.2–5.7)	0.443
% corticoid treatment	5.3	9.3	0.590
% insulin	24.5	33.8	0.203
Weeks start insulin (median)	30.0 (28.0–32.0)	29.0 (26.0–32.0)	0.367
% bolus-basal injections	17.4	52.0	**0.032**

BME: ethnic minority backgrounds; T2DM: type 2 diabetes; GDM: gestational diabetes; GCT: glucose challenge test: OGTT: oral glucose tolerance test; PCOS: polycystic ovary syndrome.

**Table 3:** Comparison of the beta-cell function and insulin sensitivity between women with a normal OGTT and women with glucose intolerance/diabetes postpartum.

	Normal *N* = 86	Glucose intolerance/diabetes *N* = 68	*P* value	Adjusted *P* value
pISSI-2 mean	1.7 ± 0.4	1.5 ± 0.5	**0.002**	**0.029**
pMatsuda (median)	2.9 (2.0–4.1)	2.7 (1.7–3.7)	0.273	0.355
p1/HOMA-IR (median)	0.02 (0.01–0.03)	0.02 (0.01–0.03)	0.204	0.641
dISSI-2 (median)	2.2 (1.8–2.6)	1.5 (1.2–1.9)	**<0.0001**	**0.020**
dInsulinogenic index/HOMA-IR (median)	0.013 (0.010–0.019)	0.008 (0.005–0.014)	**<0.0001**	0.479
dMatsuda (median)	6.0 (4.3–8.8)	3.8 (2.6–6.2)	**<0.0001**	**0.021**
d1/HOMA-IR (median)	0.036 (0.027–0.056)	0.026 (0.018–0.052)	**0.013**	0.901

Only women with complete data on glucose and insulin levels were included in the analysis. pISSI-2: insulin secretion sensitivity index during pregnancy; pMatsuda: insulin sensitivity index of Matsuda during pregnancy; p1/HOMA-IR: the reciprocal of the homeostasis model assessment of insulin resistance during pregnancy; dISSI-2: insulin secretion sensitivity index postpartum; dMatsuda: insulin sensitivity index of Matsuda postpartum; dInsulinogenic index/HOMA-IR is a measure for beta-cell function postpartum; d1/HOMA-IR: the reciprocal of the homeostasis model assessment of insulin resistance postpartum; the *P* values for the measurements during pregnancy are adjusted for age, BMI, ethnic background, multiparity, and corticoid treatment; the *P* values for the measurements postpartum are adjusted for age, BMI, ethnic background, breastfeeding, and the progestin-only oral contraceptive.

**Table 4:** Comparison of characteristics between women with an impaired fasting glucose (IFG) and women with an impaired glucose tolerance (IGT) postpartum.

	IFG *N* = 14 (21.2%)	IGT *N* = 44 (66.7%)	*P* value
Age mean years	31.4 ± 4.4	33.1 ± 4.6	0.244
BMI Kg/m² at first prenatal visit (median)	29.8 (26.5–34.7)	25.4 (22.4–28.9)	**0.012**
% overweight at first prenatal visit	35.7	45.2	0.073
% obese at first prenatal visit	50	14.3	**0.006**
% excessive weight gain	42.9	20.9	0.106
% BME	50.0	40.9	0.550
% first degree family member with T2DM	21.4	13.6	0.769
% history of GDM	35.7	9.1	0.047
% multiparous	50.0	36.4	0.592
% breastfeeding	71.4	75.0	0.225
% progestin-only oral contraceptive	66.7	71.4	0.756
Result GCT mg/dL (median)	164.0 (155.0–166.0)	170.0 (152.5–193)	0.206
Week at diagnosis of GDM (median)	25.0 (20.5–27)	27.0 (25.0–28.7)	**0.029**
% fasting abnormal 100 g OGTT	83.3	34.9	**0.003**
% ≥4 values abnormal 100 g OGTT	41.7	14.0	**0.034**
Median fasting 100 g OGTT mg/dL	99.5 (96.2–121.0)	89.0 (84.0–101.0)	**0.022**
Median 1 h 100 g OGTT mg/dL	202.5 (183–226.2)	189.0 (170.0–202.0)	**0.047**
Median 2 h 100 g OGTT mg/dL	187.5 (157.2–228.5)	175.0 (162.0–196.0)	0.554
Median 3 h 100 g OGTT mg/dL	147.5 (110.5–181.5)	152.0 (139.0–163.0)	0.927
% corticoid treatment	0	9.1 (4)	0.242
% insulin	42.9	29.5	0.355
Weeks start insulin (median)	24.0 (20.7–28.2)	30.0 (27.5–32.0)	**0.017**
% bolus-basal injections	66.7	38.5	0.412

8 Women (12.1%) with the combination of IFG and IGT were excluded from these analyses. BME: ethnic minority backgrounds; T2DM: type 2 diabetes; GDM: gestational diabetes; GCT: glucose challenge test; OGTT: oral glucose tolerance test.

**Table 5:** Comparison of the beta-cell function and the insulin sensitivity between women with an impaired fasting glucose (IFG) and women with an impaired glucose tolerance (IGT) postpartum.

	IFG *N* = 14 (21.2%)	IGT *N* = 44 (66.7%)	*P* value	Adjusted *P* value
pISSI-2 mean	1.3 ± 0.6	1.5 ± 0.4	0.211	0.927
pMatsuda (median)	2.2 (1.2–3.0)	3.0 (1.9–3.7)	0.132	0.695
p1/HOMA-IR (median)	0.013 (0.010–0.018)	0.022 (0.013–0.034)	0.070	0.813
dISSI-2 (median)	1.4 (1.2–2.2)	1.7 (1.4–1.9)	0.221	0.629
dInsulinogenic index/HOMA-IR (median)	0.005 (0.002–0.009)	0.010 (0.007–0.015)	**0.003**	0.087
dMatsuda (median)	3.2 (1.6–3.8)	4.8 (3.1–7.7)	**0.004**	0.075
d1/HOMA-IR (median)	0.020 (0.015–0.022)	0.038 (0.021–0.062)	**<0.0001**	**0.016**

pISSI-2: insulin secretion sensitivity index during pregnancy; pMatsuda: insulin sensitivity index of Matsuda during pregnancy; p1/HOMA-IR: the reciprocal of the homeostasis model assessment of insulin resistance during pregnancy; dISSI-2: insulin secretion sensitivity index postpartum; dMatsuda: insulin sensitivity index of Matsuda postpartum; dInsulinogenic index/HOMA-IR is a measure for beta-cell function postpartum; d1/HOMA-IR: the reciprocal of the homeostasis model assessment of insulin resistance postpartum; the *P* values for the measurements during pregnancy and postpartum are adjusted for BMI.
